# Reduced chemical application with bio-organic fertilizer to improve grain filling characteristics and fertilizer partial productivity of highland barley under irrigation regimes

**DOI:** 10.1371/journal.pone.0332951

**Published:** 2026-05-12

**Authors:** Yinping Xu, Xingroong Wang, Jianhua Liu, Yanjun Zhang, Kecang Huo, Hong Chang

**Affiliations:** 1 Institute of Industrial Crops and Malting Barley, Gansu Academy of Agricultural Sciences, Lanzhou, Gansu, China; 2 Institute of Crop, Gansu Academy of Agricultural Sciences, Lanzhou , Gansu, China; 3 Gansu Academy of Agricultural Sciences, Lanzhou , Gansu, China; Government College University Faisalabad, PAKISTAN

## Abstract

Reduction of nitrogen fertilizer and simultaneously application of bio-organic fertilizer is regarded as an essential approach for realizing sustainable agricultural development. Substituting partial chemical N with bio-organic fertilizer is an environmentally friendly; reduce the risk of environmental pollution due to N losses. However, the appropriate rate of bio-organic fertilizer of highland barley in China is unknown. To clarify the effect of reducing chemical fertilizer and adding bio-organic fertilizer on the grain filling characteristics and yield of highland barley after anthesis under different deficit irrigation amounts in Hexi irrigation area. Under the field test environment for two consecutive years, the differences in grain dry weight after anthesis, grain filling rate, fertilizer partial productivity and yield of highland barley were determined by irrigating once (W1) and twice (W2) during the whole growth period under the no fertilizer T1, N, P, K recommended fertilization T2, T3, T4 and T5 (the total amount of N, P, K recommended fertilizer was reduced by 15%, 30%, and 45%, and bio-organic fertilizer was increased by 30%, 60% and 90%, respectively. Grain filling under different fertilization treatments the filling duration of W2 was extended by 3–8 days compared with W1, and the grain dry weight was higher 25 days after anthesis. The maximum and average filling rates of T3 and T4 were large, the filling duration was short, and the filling rate in each period was significantly positively correlated with the thousand-grain weight. Under the recommended fertilization level T2, there is a large improvement in the partial productivity of highland barley. When the amount of chemical fertilizer was reduced by 45% and organic fertilizer was applied, the partial productivity of chemical fertilizer increased significantly. The average yield of each fertilization treatment with W2 increased by 7.48% in (2019) and 7.06% in (2020) compared with W1. Under the same irrigation, the yield of T4 was always significantly increased compared with other fertilization treatments. The results show that on the basis of the current recommended fertilization level (T2), reducing chemical fertilizer by 15% to 30%, and applying bio-organic fertilizer by 30% to 60%, the T3 and T4, can significantly increase the grain filling rate of highland barley, shorten the filling duration, and increase yield. It is also the optimized water and fertilizer management mode for highland barley cultivation regions. This study serves as an important reference for the optimal management of N fertilizer with irrigation regimes and the promotion of sustainable agricultural development of highland barley cultivation.

## 1. Introduction

Chemical fertilizer is known as the “grain” of grain [1]. Its use has greatly increased crop yield with a 57% improvement [2] and food security. However, excessive application of chemical fertilizer is common in China, and high levels of chemical fertilizer application are associated with serious problems such as soil degradation and environmental pollution [3–5]. Reducing chemical fertilizer use and increasing Bio-organic fertilizer application seems to be the inevitable path forward in agriculture in China [6–7]. Combined application of organic and in bio-organic fertilizers can balance soil nutrients and improve soil structure. This strategy improves soil microbial communities (in both quantity and structure); changes enzyme activities in the rhizosphere; reduces soil nutrient loss, soil salinization [8–11], and the occurrence of soil-borne diseases; and increases plant fertilizer use efficiency, soil organic matter content, root activity, antioxidant enzyme activity, and ultimately crop yield and stress resistance [12–15]. Furthermore, it is an effective way to implement the policy of double reduction of chemical fertilizers and pesticides, and to realize zero-growth of chemical fertilizer use. It is also rich in calcium, magnesium, phosphorus, zinc, manganese, selenium, and other micronutrients. In highland barley [16,17], the β-glucan content is highest in the grains. It is also rich in phenolic substances such as Maternal Tocopherol, flavone, and anthocyanin. These substances are natural antioxidants and have many benefits to humans: anti-cancer and anti-aging effects, prevention of cardiovascular disease, immune system improvement, and other unique physiological effects. In recent years, a great deal of attention has been paid to the health benefits of highland barley and to analysis and identification of its nutritional components and potential medicinal functions. However, most highland barley planting areas are located in the “three regions and three states” with deep poverty. Highland barley development has long been limited due to the low economic levels in those regions. There is a lack of scientific, standardized planting techniques, and there are issues such as the coexistence of excessive fertilization and nutrient deficiency; insufficiency of bio-organic fertilizer; and inattention to the law of diminishing returns with respect to fertilizer input. Therefore, it is of great significance to establish scientific fertilization procedures for highland barley. Researching cultivation techniques that reduce chemical fertilizer use and increase bio-organic fertilizer application to highland barley will minimize environmental pollution and promote green agricultural development [18,19].

In recent years, many scholars have done a lot of research on the effects of fertilization on barley growth and development, dry matter accumulation and distribution, and yield formation. Huiquan et al. [17] believed that the nitrogen fertilizer base-topdressing ratio was 7:3 when the dry matter transport, contribution rate to grains and grain yield of barley after flowering reached the maximum. Xu et al. [20] and Zhao et al. [21] established that the nitrogen application rates when the dry matter accumulation of each organ of barley after flowering was the highest and the contribution rate to grains was the highest were 212.42 ~ 261.97 kg/ha and 180 kg/ha, respectively. In the study of barley filling characteristics, Liu et al. [22], Li et al. [23] and Feng et al. [24] studied the correlation between the external morphological indicators of barley plants and the characteristic parameters of barley filling. In addition, the research results of Yan Jie et al. showed that soil water stress reduced the filling rate of barley grains and shortened the filling period [25,26]. Wang et al. [27] believed that the nitrogen fertilizer level had little effect on the grain mass increase process of two barley varieties in the early filling period. The grain mass of the treatment without nitrogen fertilizer after the filling peak was significantly higher than that of the treatment with nitrogen fertilizer. After 30 days of flowering, the grain filling rate gradually increased with the increase of nitrogen application. Zhu et al. [28] showed that QTB13 and Zangqing 27 took the longest time to reach the maximum filling rate. The accumulation amount, theoretical maximum thousand-grain mass, accumulation amount in the gradual increase stage, duration of the rapid increase stage, accumulation amount, and fertilizer level at the maximum thousand-grain weighy were 90 kg/ha and 0 kg/ha, respectively.

This study conducted a trial study on the cultivation technology of highland barley with reduced chemical fertilizer and combined application of bio-organic fertilizer in the Hexi irrigation area. By measuring and analyzing the index parameters such as dry matter accumulation of highland barley after flowering, filling characteristics, grain yield, and fertilizer utilization efficiency, the optimal ratio of reduced chemical fertilizer and combined application of bio-organic fertilizer in this area was clearly proposed, which is of great significance to scientific fertilization of highland barley production and the promotion of green agricultural development.

## 2. Materials and Methods

### 2.1. Plant materials and fertilizers

**Fig 1:**
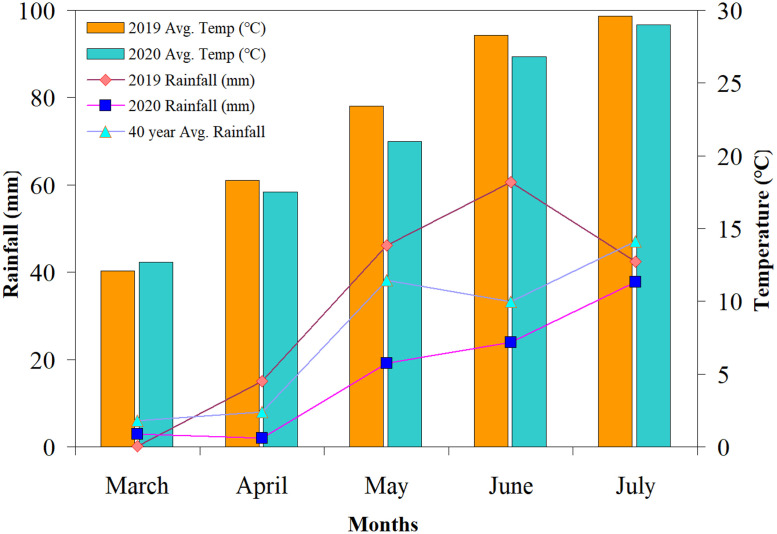
Monthly rainfall and temperature distribution during the barley growing seasons in 2019 and 2020 study years.

The barley variety ‘Longqing 1’ was used for this study. ‘Longqing 1’ is a new spring highland barley variety that was bred by the Gansu Academy of Agricultural Sciences. The chemical fertilizer used contained urea (N, 46%) and single super phosphate (phosphorus 14.5%). The bio-organic fertilizer had a total nutrient content of N + P_2_O_5_ + K_2_O ≥ 5.0%, organic matter ≥ 45.0%, potassium humate ≥ 30%, and silicon, calcium, and magnesium ≥ 40%. The bio-organic fertilizer was produced by the Beijing Yuntianhua Tri-ring Fertilizer Co., Ltd. Fig 1 shows the monthly precipitation and temperature distributions during the two-year barley field study.

### 2.2. Field experiments

Field experiments were carried out from 2019 to 2020 at the wheat comprehensive experimental station of Gansu Academy of Agricultural Sciences in Huangyang, Liangzhou District, Wuwei City, Gansu Province, and China. This region is located in the east of the Hexi Corridor of Gansu Province, which is a continental temperate zone with an arid and semi-arid climate. There is an annual average of 2360–2920 sunshine hours, 135–150 frost-free days, 210 mm rain, and 2019 mm evaporation; the average elevation is 1766 m, and the annual average temperature is 6.5°C. The test site contained irrigated desert soil. Before the test, soil samples were collected at multiple points from 0–20 cm of the arable layer. The soil composition was as follows: 0.90 g/kg total nitrogen, 0.46 g/kg total phosphorus, 24.21 mg/kg total potassium, 85.16 g/kg alkaline hydrolyzed nitrogen, 16.66 mg/kg available phosphorus, 197.67 mg/kg available potassium, and 30.14 mg/kg organic matter. The pH was 8.27.

A split-plot design was adopted for the experiment with three replications. There were two irrigation treatments, W1 and W2. Plants in the W1 treatment group were irrigated once during the jointing period (on May 3) at a rate of 1500 m^3^/ha. Plants in the W2 treatment group were irrigated once at the jointing stage (on May 3) and once at the flowering stage (on June 22) at a rate of 1500 m^3^/ha. Plants had one of five fertilizer treatments applied: T1 was the no fertilizer control (CK); T2 had the recommended levels of N, P, and K (120 kg Nha^-1^, 106 kg Pha^-1^, and 0 kg Kha^-1^); T3 had 85% of the recommended N, P, and K levels with 30% bio-organic fertilizer; T4 had 70% of the recommended N, P, and K levels with 60% bio-organic fertilizer; and T5 had 55% of the recommended N, P, and K levels with 90% bio-organic fertilizer as show in (Table 1). The plot area was 25 m^2^ (5.0 × 5.0 m), with 0.25 cm between rows. Twenty rows were planted with 560 seeds each on March 18, 2019 and March 19, 2020. Seeds were planted via manual ditching and spreading, which was repeated three times. Each fertilizer treatment was evenly applied as a dressing fertilizer to the appropriate plot one time. Irrigation was measured with a water meter. Plots were covered with transparent plastic films which are deep into 50 cm to prevent leaching into other fields. Other parameters were managed uniformly based on local practices.

**Table 1:** Details of experimental treatments.

NO.	Treatment	Specific fertilizer amount
T1	CK	No fertilizer is applied
T2	N, P, K recommended formula fertilizer total amount	120 kg N ha^-1^, 106 kg P ha^-1^, 0 kg K ha^-1^
T3	N, P, K recommended formula fertilizer amount is reduced by 15% + 30% bio-organic fertilizer	102 kg N ha^-1^ + 90.1 kg P ha^-1^+ 0 kg K ha^-1^ + bio-organic fertilizer 450 kg ha^-1^
T4	N, P, K recommended formula fertilizer amount is reduced by 30% + 60% bio-organic fertilizer	84 kg N ha^-1^ + 74.2 kg P ha^-1^ + 0 kg K ha^-1^ + bio-organic fertilizer 900 kg ha^-1^
T5	N, P, K recommended formula fertilizer total amount reduced by 45% + 90% bio-organic fertilizer	66 kg N ha^-1^ + 58.3 kg P ha^-1^ + 0 kg K ha^-1^ + bio-organic fertilizer 1350 kg ha^-1^

In this study, T2 is the reference point for the recommended fertilizer application rate for chemical fertilizers, and the reference point for the recommended fertilizer application rate for bio-organic fertilizers is 1500 kg ha^-1^.

### 2.3. Analysis of yield and grain filling rate

Each plot was harvested and dried separately to measure yield. After sun drying, grain yield was calculated based on 13.0% water content. The flowering date of each treatment was selected as the same date of flowering, well-developed and evenly-sized spikes and tag. Starting on the day of flowering (0 d), 10 plants were sampled every 5 d until plants reached full ripeness. Tassel weight was measured, and then 10 grains were peeled from the middle of each tassel. One hundred grains were heated at 105°C for 30 min, and then dried at 80°C to a constant weight. The filling rate was calculated as the weight per grain per day in mg per grain per day, and then the change in grain weight was fitted with a logistic equation.

The grain filling process was fitted with a logistic equation as follows [29]:


W = K / (1 + eA + Bt)

where t was the time after anthesis, W was the corresponding grain dry weight at that time, A and B were the parameters determined by the equation for different varieties, k (mg/grasin) was the highest grain mass in fitting theory, and e was the base of the natural logarithm function. The equation of the grain filling rate was obtained from the first derivative of this equation, and the following characteristic parameters of grain filling were obtained:

Initial potential of grain growth: C0 = K / (1 + eA) [30]

Time at maximum grain filling rate: Tmax(d) = −A / B [31]

Maximum grain filling rate: Max (mg / (D)) = −KB / 4 [30]

Filling duration: T(d) [29]

Average grain filling rate: R_mean_(mg/ (grain· d)) = grain weight gain/filling duration [30].

The infilling rate curve had two inflection points. The first derivative of the infilling rate equation was used to obtain the values (t1 and t2) of the inflection points in the T coordinate. To allow t3 to reach 96% of the time to maximum grain weight, the lengths of three stages of the filling process were determined, namely the grain-filling time during the grain-filling pyramid period (T1), the grain-filling time during the grain-filling fast increase period (T2), and the grain-filling time during the grain-filling slow increase period (T3). The increased grain weight at each stage was recorded as W1, W2, and W3, respectively; R1, R2, and R3 are the three different corresponding grain filling periods, respectively.

### 2.4. Analysis of nutrient partial productivity

Nutrient partial productivity (PFP) and partial fertilizer productivity (PFPT) was calculated as follows [32]:


PFP=Yd/F

where Y was the highland barley yield per unit area (kg/ha) and F was the total fertilizer application (kg/ha). Chemical fertilizer partial productivity (PFPC) was calculated as follows:


PFPC = Y/C

where Y was the highland barley yield per unit area (kg/ha) and C was the amount of fertilizer applied (kg/ha).

### 2.6. Statistics analysis

SPSS 18.0 (SPSS Inc., Chicago, IL, USA) was used to analyze the data. Microsoft Excel 2013 software was used for statistical mapping. If the F-test is significant, evaluate the mean using the (LSD 0.05) multiple comparison test.

## 3. Results

### 3.1. Variations and differences in 1000-grain weight after anthesis

There were significant differences in 1000-grain weight between time points after anthesis in both study years (Table 2). In the within-year comparisons, the average grain weight was higher at each post-anthesis time point for plants under W2 irrigation than that of W1 irrigation. Under W1 irrigation in both study years, the 1000-grain weight at 15–25 d after anthesis was close to 50% of the grain dry weight from the whole filling duration, and the plumpness reached 52.66–88.63% in 2019 and 53.34–89.32% in 2020. Under W2 irrigation, the 1000-grain weight at 15–30 d after anthesis was close to 50% of the dry weight from the whole filling duration, and the plumpness was 54.99–94.38% in 2019 and 54.33–97.30% in 2020. These results showed that 15–25 d was the key 1000-grain weight for plants in the W1 irrigation group, whereas 15–30 d was the key 1000-grain weight for plants in the W2 group. These two time periods thus greatly affected the final 1000-grain weight and plumpness in highland barley.

**Table 2:** Comparison of thousand grain weight after anthesis.

Year	Treatments	1000-grain weight (g)													
		5d		10d		15d		20d		25d		30d		35d	
		W1	W2	W1	W2	W1	W2	W1	W2	W1	W2	W1	W2	W1	W2
2019	T1	4.19c	4.29c	7.01d	10.22b	13.40d	16.46b	20.07c	21.46b	24.26 c	27.13c	28.17d	31.29b	30.62c	34.89b
	T2	5.13b	6.73ab	10.69c	13.21ab	20.17c	22.33ab	29.66b	31.57ab	36.41b	38.96abc	39.33bc	41.37ab	41.26ab	43.13ab
	T3	7.47a	7.02a	13.21ab	16.74a	24.42b	28.28a	35.94a	35.19a	40.34ab	40.48a	41.83ab	42.33a	43.27ab	44.24ab
	T4	7.74a	7.88ab	15.09 a	17.06ab	27.56a	29.15ab	37.21a	38.06a	41.78a	43.53ab	43.86a	46.37a	45.76a	48.29a
	T5	7.33a	6.96a	11.11bc	11.29b	20.49c	20.01b	31.67b	29.19ab	35.66b	33.53bc	37.73c	37.72ab	39.23b	40.39ab
Analysis of variance															
	W	*		*		*		*		*		*		*	
	F	*		*		*		*		*		*		*	
	W x F	ns		ns		ns		ns		ns		ns		ns	
2020	T1	4.24c	6.82c	7.09d	12.35b	13.92 d	17.51c	20.02d	25.48c	23.18c	31.35b	25.81c	35.58b	26.94c	36.27b
	T2	5.93b	7.68b	11.61c	18.83a	20.97bc	23.70bc	31.90bc	31.36ab	37.93a	40.51ab	41.20a	47.83ab	42.58a	47.11ab
	T3	7.99a	9.27ab	14.03b	20.23a	23.19b	29.40ab	33.81ab	40.11a	41.15a	48.39a	43.34a	51.52ab	44.49a	52.71a
	T4	8.20a	9.93a	17.27a	21.83a	27.04a	32.11a	37.09a	43.08a	42.12a	51.26a	45.01a	53.21a	46.30a	55.37ab
	T5	8.02a	9.13b	9.91c	18.27b	19.71c	28.14bc	27.64c	36.13bc	31.18b	43.03b	34.31b	46.19ab	36.25b	49.37ab
Analysis of variance															
	W	*		*		*		*		*		*		*	
	F	*		*		*		*		*		*		*	
	W x F	ns		ns		ns		ns		ns		ns		ns	

T1, no fertilization; T2, fertilization with the recommended nitrogen (N), phosphorus (P), and potassium (K); T3, fertilization with 85% of the recommended N/P/K formula and 30% bio-organic fertilizer; T4, fertilization with 70% of the recommended N/P/K formula and 60% bio-organic fertilizer; T5, 55% of the recommended N/P/K formula and 90% bio-organic fertilizer. Lowercase letters after numbers within a column indicate statistically significant differences at p < 0.05.

**Fig 2:**
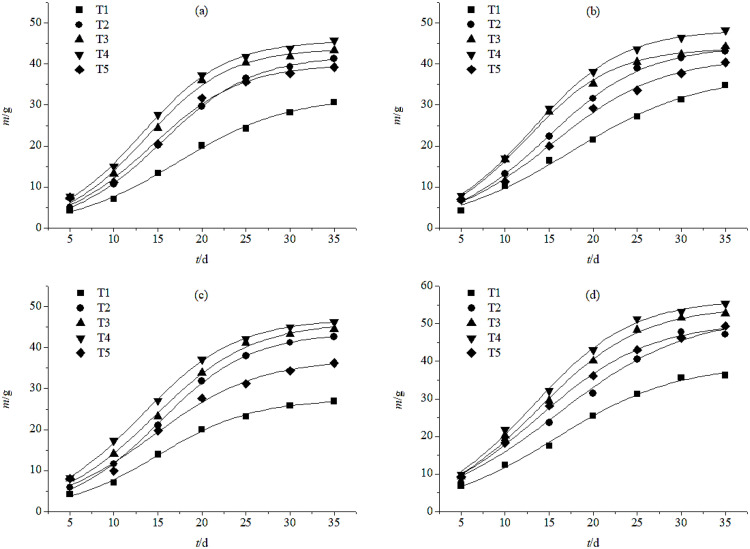
Grain dry weight after anthesis for plants irrigated once (a & c) and plants irrigated twice (b & d) under different fertilizer treatment in 2019 (a & b) and 2020 (c & d). T1, no fertilization; T2, fertilization with the recommended nitrogen **(N)**, phosphorus **(P)**, and potassium **(K)**; T3, fertilization with 85% of the recommended N/P/K formula and 30% bio-organic fertilizer; T4, fertilization with 70% of the recommended N/P/K formula and 60% bio-organic fertilizer; T5, 55% of the recommended N/P/K formula and 90% bio-organic fertilizer.

There were significant differences in grain dry weight between fertilization treatments, but not between irrigation treatments over the two years. The grain dry weight of plants in the T2, T3, T4 and T5 groups were significantly higher than those in the T1 group under both irrigation treatments at different times of year. The difference in grain dry weight between fertilization treatments was also significant between irrigation treatments, but the results were not consistent between years. The grain dry weight of plants in the T3 and T4 treatments were higher than those of T1 plants in both irrigation groups in both years. Under W2 irrigation, the average grain dry weight was similar between T2- and T5-treated plants until 15 d after anthesis; grain dry weight was lower in T2- than T5-treated plants between 15 and 25 d after anthesis, but there was no significant difference between T2 and T5 grain dry weight between 30 and 35 d after anthesis. Thus, from 0–35 d after anthesis, grain weight showed an increasing trend; grain weight rapidly increased in the early stage of grain filling, then the growth rate gradually decreased in the late stage (Fig 2). Under both W1 and W2 irrigation, the grain weight was higher in both years than that of other fertilization treatments at the early filling stage, and in the medium-term, grains enriched quickly; the grain weight was 41.78–51.26 g at 25 d after anthesis, and 43.0 g or heavier at 30 d. In the W2 group in 2020, the grain dry weight of plants in the T4 group was measured as high as 53.21 g at 30 d after anthesis, which was close to the final grain weight.

### 3.2. Grain filling rate after anthesis

**Fig 3:**
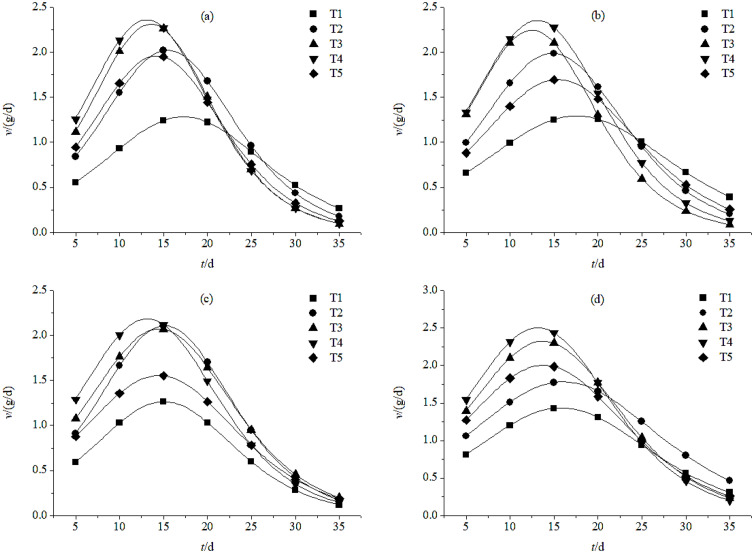
Grain filling rate after anthesis for plants irrigated once (a & c) and plants irrigated twice (b & d) under different fertilizer treatment in 2019 (a & b) and 2020 (c & d). T1, no fertilization; T2, fertilization with the recommended nitrogen **(N)**, phosphorus **(P)**, and potassium **(K)**; T3, fertilization with 85% of the recommended N/P/K formula and 30% bio-organic fertilizer; T4, fertilization with 70% of the recommended N/P/K formula and 60% bio-organic fertilizer; T5, 55% of the recommended N/P/K formula and 90% bio-organic fertilizer.

In both the W1 and W2 groups, the post-anthesis grain filling rate of plants showed a single peak curve (Fig 3) in all five fertilization treatment groups in both years. There were between-year differences in grain filling rate among plants in the same irrigation treatment groups, and there were within-year differences between plants in different irrigations groups. However, in all cases, the grain filling rate peaked between 10 and 20 d after anthesis. In both irrigation groups, T4 had the highest and T1 had the lowest grain filling rate. In 2019, plants in the W1 and W2 groups had peak rates of 2.36 mg/(grain·d) and 2.35 mg/(grain·d), respectively, in T4-treated plants. In 2020, the values for the same treatment groups were 2.18 mg/(grain·d) and 2.50 mg/(grain·d), respectively. The peak value of the T1 group was < 1.43 mg/(grain·d) between years and irrigation groups; this was 1.08 mg/(grain·d), 1.06 mg/(grain·d), 0.91 mg/(grain·d), and 1.07 mg/(grain·d) lower than the values for the corresponding T4 groups. For both irrigation treatments in 2019 and for W1 in 2020, the order of fertilizer treatment groups from highest to lowest grain filling rate was as follows: T4, T3, T2, T5, and T1. For W2 irrigation in 2020, the order was reversed for T2 and T5, but the order of the other groups remained the same. Under both irrigation treatments, T4- and T3-treated plants had higher grain filling rates and 1000-grain weight after anthesis, specifically at 20 d. In contrast, the grain filling rates of T1 and T5-treated plants were lower after anthesis, which resulted in low 1000-grain weight.

### 3.3. Grain filling characteristics

A logistic equation was used to fit the grain filling process for the two-year experiment. The coefficient of determination (R^2^) ranged from 0.9755 to 0.9997 (Table 3), which indicated that the equation could accurately reflect the grain filling process of highland barley under different fertilization and irrigation treatments. The whole grouting process can be divided into three stages: grain-filling time during the grain-filling pyramid period (T1), grain-filling time during the grain-filling fast increase period (T2), and grain-filling time during the grain-filling slow increase period (T3). Seed dry weight slowly increased from 0 to 15 d after anthesis. The grain filling duration was longer in 2020 than in 2019, by 1.55 d and 2.92 d for W1 and W2, respectively. In both years, W2 had a longer grain filling period than W1, by 2.65 d in 2019 and 4.02 d in 2020. The time period from 15–25 d after anthesis was the key grain filling period, during which time the grain dry weight increased rapidly. The duration of grain filling varied between years and irrigation treatment groups, but the difference was < 1 d. From 25 d after anthesis to maturity, the grain dry weight increased slowly then stabilized. The duration of that period was also different between years and irrigation treatment groups; under W2 irrigation, it was longer by 2.91 d (2019) and 8.11 d (2020) compared to W1. The grouting (T) duration was longer in 2020 than in 2019, by 2.57 d (W1) and 5.45 d (W2). In the same year, W2 was longer than W1, by 5.23 d (2019) and 8.14 d (2020). These results demonstrate that irrigation had a greater effect on the duration of grain filling than fertilization did.

**Table 3:** Characteristic parameters in grain filling stage of all treatments under different irrigation volume.

Year	Treatments	R_max_		T_max_		R_mean_		T1		T2		T3		R1		R2		R3		T		R^2^	
		W1	W2	W1	W2	W1	W2	W1	W2	W1	W2	W1	W2	W1	W2	W1	W2	W1	W2	W1	W2	W1	W2
2019	T1	1.28	1.29	17.26	17.58	0.29	0.29	11.25	5.47	9.03	8.04	25.38	29.52	0.32	0.32	1.13	1.13	1.11	1.12	57.01	66.10	0.9968	0.9923
	T2	2.02	1.99	15.46	14.83	0.45	0.44	8.25	10.16	8.62	7.47	33.16	35.03	0.50	0.49	1.77	1.74	1.76	1.73	47.41	50.98	0.9997	0.9991
	T3	2.31	2.25	13.58	12.49	0.52	0.50	8.01	9.79	7.35	6.06	34.51	34.59	0.57	0.56	2.03	1.97	2.01	1.95	43.18	44.56	0.9946	0.9962
	T4	2.36	2.35	13.10	13.11	0.53	0.53	8.99	10.33	6.73	6.35	36.06	28.13	0.58	0.58	2.07	2.06	2.05	2.04	44.17	46.88	0.9982	0.9988
	T5	1.96	1.70	14.23	15.45	0.44	0.38	9.01	12.50	7.53	7.38	31.51	32.79	0.49	0.42	1.72	1.49	1.70	1.47	46.49	55.90	0.9894	0.9956
Analysis of variance																							
	W	*		*		*		*		*		*		*		*		*		*		*	
	F	*		*		*		*		*		*		*		*		*		*		*	
	W x F	ns		ns		ns		ns		ns		ns		ns		ns		ns		ns		ns	
2020	T1	1.27	1.43	15.02	15.80	0.28	0.32	9.70	15.38	7.88	6.80	21.64	30.91	0.31	0.36	1.11	1.26	1.10	1.25	49.44	62.37	0.9962	0.9956
	T2	2.07	1.78	15.14	16.03	0.47	0.40	8.45	17.43	8.33	6.38	34.37	41.21	0.52	0.44	0.85	0.56	1.83	1.55	47.17	66.92	0.9989	0.9755
	T3	2.11	2.32	14.53	13.75	0.46	0.52	10.90	13.05	7.19	6.01	36.43	43.04	0.51	0.58	1.82	2.04	1.82	2.02	50.94	53.59	0.9964	0.9963
	T4	2.18	2.50	13.15	13.16	0.49	0.56	11.40	12.60	6.07	5.73	37.08	44.53	0.54	0.62	1.92	2.19	1.90	2.17	49.11	51.51	0.9992	0.9965
	T5	1.56	2.00	14.48	13.81	0.35	0.45	12.79	14.89	6.61	5.53	29.32	39.72	0.39	0.50	1.36	1.76	1.35	1.74	54.53	57.40	0.9838	0.9974
Analysis of variance																							
	W	*		*		*		*		*		*		*		*		*		*		*	
	F	*		*		*		*		*		*		*		*		*		*		*	
	W x F	ns		ns		ns		ns		ns		ns		ns		ns		ns		ns		ns	

T1, no fertilization; T2, fertilization with the recommended nitrogen (N), phosphorus (P), and potassium (K); T3, fertilization with 85% of the recommended N/P/K formula and 30% bio-organic fertilizer; T4, fertilization with 70% of the recommended N/P/K formula and 60% bio-organic fertilizer; T5, 55% of the recommended N/P/K formula and 90% bio-organic fertilizer. Lowercase letters after numbers within a column indicate statistically significant differences at p < 0.05. R_max_, maximum grain-filling rate (mg/(grain·d)); T_max_, days to reach the maximum grain-filling rate; R_mean_, mean grain filling rate (mg/(grain·d)); T1, grain-filling time during the grain-filling pyramid period (in days); T2, grain-filling time during the grain-filling fast increase period (in days); T3, grain-filling time during the grain-filling slow increase period (in days); R1, grain-filling rate during the grain-filling pyramid period (mg/(grain·d)); R2, grain-filling rate during the grain-filling fast increase period (mg/(grain·d)); R3, grain-filling rate during the grain-filling slow increase period (mg/(grain·d)); T, grain-filling time (in days); R^2^, coefficient of determination.

### 3.4. Correlation between grain filling parameters and 1000-grain weight

The correlations between 1000-grain weight and characteristic parameters of grain filling were next analyzed (Table 4). Several parameters had extremely significant or significant positive correlations with 1000-grain weight: the maximum grain-filling rate period (R_max_); the mean grain-filling rate (R_mean_); the grain-filling rate during the grain-filling pyramid period (R1); the grain-filling rate during the grain-filling fast increase period (R2); and the grain-filling rate during the grain-filling slow increase period (R3). In contrast, several other parameters were negatively correlated with 1000-grain weight: time to maximum filling rate (T_max_); the grain-filling time during the grain-filling fast increase period (T2); the grain-filling time during the grain-filling slow increase period (T3); and the filling duration (T). These results indicated that filling rate had a greater effect than filling duration on grain weight during the whole filling period of highland barley.

**Table 4:** Correlation coefficient between grain filling parameters and 1000-grain weight.

Year Year	R_max_		T_max_		R_mean_		T1		T2		T3		R1		R2		R3		T	
	W1	W2	W1	W2	W1	W2	W1	W2	W1	W2	W1	W2	W1	W2	W1	W2	W1	W2	W1	W2
2019	0.614*	0.620*	−0.644**	−0.518*	0.663**	0.616*	−0.119	0.593*	−0.453	−0.444	−0.694**	−0.129	0.590*	0.621*	0.589*	0.659**	0.551*	0.625*	−0.601*	−0.612*
2020	0.534*	0.694*	−0.578*	−0.330	0.604*	0.729**	0.587*	−0.033	−0.711**	−0.280	−0.599*	−0.057	0.543*	0.557*	0.654**	0.730**	0.546*	0.540*	−0.277	−0.151

* and ** indicate significant differences at 0.05 and 0.01 levels. The same as below

### 3.5. Effects of irrigation and fertilizer treatments on highland barley partial factor productivity from applied fertilizer

**Fig 4:**
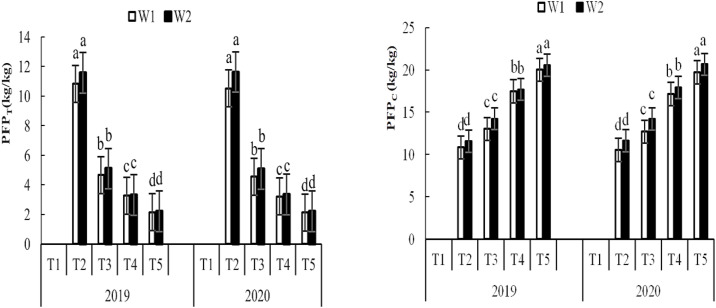
Effects of irrigation volume and fertilizer application on highland barley partial factor productivity from applied fertilizer during 2019 and 2020 study year. T1, no fertilization; T2, fertilization with the recommended nitrogen **(N)**, phosphorus **(P)**, and potassium **(K)**; T3, fertilization with 85% of the recommended N/P/K formula and 30% bio-organic fertilizer; T4, fertilization with 70% of the recommended N/P/K formula and 60% bio-organic fertilizer; T5, 55% of the recommended N/P/K formula and 90% bio-organic fertilizer. Different small letters on each column graph indicate significant differences among the treatments at 0.05 level. The same below.

In 2019 and 2020, there were significant differences in fertilizer partial productivity (PEPT) between fertilization treatment groups. There was no significant difference in PEPT between irrigation groups for T1-treated plants, which had no fertilizer added (Fig. 4). PEPT was significantly higher for T2-treated plants than for the other fertilization treatments. Under W1 irrigation, PEPT increased by 133.14%, 252.08%, and 215.33% in T2-treated plants compared to T3-, T4-, and T5-treated plants, respectively, in 2019; in 2020 under the same conditions, the increases in T2 were 131.31%, 227.93%, and 596.25%, respectively. Under W2 irrigation, PEPT was higher in T2 plants by 127.14%, 249.83%, and 423.25% in 2019 and by 128.60%, 247.04%, and 423.31% in 2020 in T3-, T4-, and T5-treated plants, respectively.

Experimental data from both years (Fig 4) showed that PFPC was highest in T5, second-highest in T4, third-highest in T3, and lowest in T2 and T1 (dependent on irrigation conditions); differences between treatment groups were significant. The results showed that PEPT was greatly improved in highland barley by treatment with the current recommended fertilizer standards (treatment T2), and that PEPT increased significantly with a 45% reduction in the recommended chemical fertilizer level combined with Bio-organic fertilizer application. This clearly demonstrated that a reduction in chemical fertilizer use and supplementation with Bio-organic fertilizer could improve partial productivity and fertilizer use efficiency.

### 3.6. Effects of irrigation and fertilizer treatments on highland barley yield

**Fig 5:**
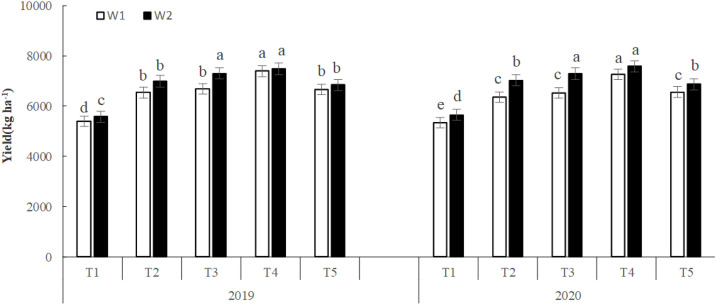
Effects of irrigation volume and fertilizer application on highland barley yield. T1, no fertilization; T2, fertilization with the recommended nitrogen **(N)**, phosphorus **(P)**, and potassium **(K)**; T3, fertilization with 85% of the recommended N/P/K formula and 30% bio-organic fertilizer; T4, fertilization with 70% of the recommended N/P/K formula and 60% bio-organic fertilizer; T5, 55% of the recommended N/P/K formula and 90% bio-organic fertilizer. Different small letters on each column graph indicate significant differences among the treatments at 0.05 levels.

Yield was higher in W2 than W1 plants in both years; the average yield was 7.48% and 7.06% higher under W2 than W1 irrigation in 2019 and 2020, respectively. For T3- and T4-treated plants, yield increased by > 10.0% under W2 compared to W1 irritation. T2-, T3-, T4-, and T5-treated plants had higher yield than T1-treated plants across years and irrigation treatment groups. Specifically, fertilized plants had higher yield by 21.28–37.03% (2019, W1), 22.755–34.35% (2019, W2), 18.18–36.18% (2020, W1), and 24.46–34.38% (2020, W2). T4-treated plants consistently had the highest yield, and the difference compared to the other fertilization treatments was statistically significant. In T4-treated plants, yield was more than 31.0% higher than in T1-treated plants. T3-treated plants also had higher yield than T2 and T1 plants, with a difference of 22% between T3- and T1-treated plants (Fig 5).

## 4. Discussion

Nitrogen is regarded as a primary factor affecting crop growth, GY, and NI, and optimizing N fertilizer management practices is essential for improving crop performance and yield. Differences in crop DMA among treatments partly explained the source of differences in crop GY and NI [33,34]. The results of this study showed that the experimental treatments T3 and T4, i.e., the application rate of chemical fertilizer per hectare was 84–102 kg N ha^-1^, 74.2–90.1 kg P ha^-1^, and 0 kg K ha^-1^, and the application rate of biological organic fertilizer was 450–900 kg/ha, which could significantly increase the aboveground biomass accumulation of highland barley at different growth stages. This phenomenon was particularly obvious in the middle and late stages of grain filling, which laid a biological basis for the increase in highland barley grain yield. This is consistent with the research results of Xu et al. [35].

Grain filling is an important physiological process in the formation of barley grains and is the key to the formation of grain weight and yield. The grain filling characteristics of barley grains are controlled by multiple factors such as genetics, environment, and water and fertilizer management. Among them, genetic factors are the main controlling factors, and fertilizer is the main environmental factor regulating grain filling. In this study, at the same fertilization level, the grain filling rate of W2 treatment was greater than that of W1, and the grain filling time was extended by 3−8 days. Since the W1 treatment was irrigated only at the jointing stage of highland barley at an irrigation rate of 1500 m^3^/ha, while the W2 treatment was irrigated at the jointing stage and flowering stage at an irrigation rate of 1500 m^3^/ha/time, compared with the W2 treatment, the W1 treatment was in a state of soil water stress in the late stage of highland barley growth, which was also the key period for the formation of highland barley grain yield, resulting in a decrease in the grain filling rate of highland barley, a shortened grain filling period, incomplete grain filling, and ultimately a decrease in highland barley grain yield. The rice cultivar “super” does not ascertain high potential yield due to poor seed filling in the upper seeds [30]. The potential yield of crop can be divided into three main components: number of spike plant-1, seeds spike-1, and seed weight. Seed filling rate, regulated by seed weight, is an essential yield component of crop [31]. An earlier study has shown that crop seed weight is significantly affected under irrigation conditions [36].

The results of this study showed that in treatments T3 and T4, that is, the fertilizer application rate per hectare was: 84–102 kg N ha^-1^, 74.2–90.1 kg P ha^-1^, and 0 kg K ha^-1^; when the bio-organic fertilizer application rate was 450–900 kg/ha, the highland barley filling rate was always higher than that of other fertilization treatments under the two irrigation rates, and its final yield and thousand-grain weight were also correspondingly higher. This is because compared with other fertilization treatments, this ratio of chemical fertilizer and biological organic fertilizer can improve the soil structure for highland barley growth, better balance soil nutrients, reduce the loss of water and nutrients in the soil, so that the soil can continuously supply the nutrients and water required for highland barley filling during the filling process, achieve full filling, and ultimately increase highland barley yield. The relatively slow N release rate from bio-organic fertilizer reduces N loss and waste, creating opportunities for long-term N uptake by plants, which ultimately facilitates continued crop growth [37,38]. Relevant studies have confirmed that treatment that replaced in whole or in part of bio-organic fertilizer produced the largest amounts of soluble phosphorus and potassium and increased grain yield compared with inorganic fertilizer applied alone [39,40].

At present, most of the research on the correlation between crop filling parameters and yield is reflected in the correlation between thousand-grain weight and filling parameters, and the conclusions vary due to different crop types, ecological zones and cultivation measures. Through correlation analysis, this study found that the maximum filling rate, average filling rate, filling rate in the gradual increase period, filling rate in the rapid increase period and filling rate in the slow increase period were all extremely significantly or significantly positively correlated with thousand-grain weight; this is basically consistent with the research results of Brdar et al. [41], Gao et al. [42] and Zhu et al. [43]. The maximum filling rate, the time of rapid filling period, the time of slow filling period and the number of days of filling were all extremely significantly, significantly or insignificantly negatively correlated with the thousand-grain weight; this is consistent with the research results of Cai et al. [44] and Luo et al. [45]. In the future, we should focus on the regulation of bio-organic fertilizer replacing partial chemical N fertilizers on soil physicochemical properties and microbial diversity in barley planting system to provide new perspectives on how reduced chemical application with bio-organic fertilizer to improve grain filling characteristics and fertilizer partial productivity of highland barley.

## 5. Conclusions

We here tested the current recommended fertilizer standards (120 kg Nha^-1^, 106 kg Pha^-1^, and 0 kg Kha^-1^) by comparing highland barley plants grown under two irrigation conditions with five fertilizer application rates. The combined chemical application with bio-organic fertilizer treatment significantly increased the grain filling rate, grain filling duration, and increased the partial productivity of chemical fertilizer under two different irrigation regimens. These results lay a biological foundation for increased highland barley grain yield, and offer an optimized irrigation and fertilizer management plan for highland barley cultivation in the region. Based on the currently recommended fertilization levels of 120 kg Nha^-1^, 106 kg Pha^-1^, and 0 kg Kha^-1^, a 15% to 30% reduction in chemical fertilizer application and a 30% to 60% application of bio-organic fertilizer can significantly increase the barley grain filling rate, grain yield and partial productivity of chemical fertilizer under both irrigation conditions. This study provides new insights into sustainable cultivation pathways for highland barley under irrigation conditions. In the future, we should focus on the regulation of bio-organic fertilizer replacing partial chemical N fertilizers on soil physicochemical properties and microbial diversity in barley planting system to provide new perspectives on how reduced chemical application with bio-organic fertilizer to improve grain filling characteristics and fertilizer partial productivity of highland barley.

## Supporting information

S1 Data Research data. (XLS)
